# Can commensals alter pathogen’s antibiotic resistance during co-culture?

**DOI:** 10.1099/jmm.0.002126

**Published:** 2026-02-24

**Authors:** Alexander D. H. Kingdon, Elena Jordana-Lluch, Kim Rachael Hardie

**Affiliations:** 1 Biodiscovery Institute and National Biofilms Innovation Centre, School of Life Sciences, University of Nottingham, Nottingham, NG7 2RD, UK

**Keywords:** antibiotic resistance, polymicrobial biofilm, *Pseudomonas aeruginosa*, *Staphylococcus*, *Micrococcus luteus*

## Abstract

**Introduction.** Bacterial infections of skin wounds can increase hospitalization duration and lead to worse patient prognoses, especially for burn wounds and diabetic foot ulcers. The two main pathogens which infect these wounds are *Pseudomonas aeruginosa* and *Staphylococcus aureus*. However, many other species can be present in wound infections, including skin commensal bacteria such as *Staphylococcus epidermidis* and *Micrococcus luteus*.

**Hypothesis.** It was hypothesized that co-infection alters the antibiotic resistance of each species present.

**Aim.** To investigate dual-species commensal-pathogen co-culture and assess the potential influence on the antibiotic resistance of each species.

**Methodology.** The commensal and pathogenic species were grown either separately or in dual-species co-culture, potentially allowing biofilm formation for 24 h and were subsequently treated with antibiotics (ciprofloxacin or tobramycin). The impact of the co-culture growth was compared with single species cultures and the effect of the antimicrobial treatment on both conditions were assessed through Minimum Biofilm Eradication Concentrations (MBECs) and bacterial viable counts.

**Results.** The viability of each bacterial species was reduced in the presence of other species, and this translated to reduced antibiotic resistance (lower MBECs) of *P. aeruginosa* in particular. The resistance of the other species appeared more dependent on the specific inter-species effects.

**Conclusion.** The inclusion of a commensal species with pathogens in co-culture reduced the antibiotic resistance, and inter-species effects influenced the viability of the pathogens. More realistic antimicrobial resistance assessment protocols accounting for microbial communities could therefore lead to more effective treatments.

## Introduction

Skin wound infections are a major class of chronic infections, which cost the UK healthcare service over £1 billion per year [1], and in 2022, the global healthcare-associated cost to chronic wounds was estimated to be $148.65 billion [2]. Due to their links to diabetes and obesity, chronic wound numbers are likely to increase in the future [3]. Wounds are described as chronic once they have not healed for over 1 month and typically contain skin commensal micro-organisms [4,5]. This delayed healing could allow several different pathogens [6,9], including antimicrobial-resistant ones [9,10], to invade the wounds. A majority of chronic infections have been reported to be polymicrobial in nature [5,11], and hence, understanding the polymicrobial interactions which are occurring [12,14] and being able to model these in a laboratory could lead to more robust treatment screening methods.

Two pathogenic species are predominantly found in wound infections: *Staphylococcus aureus* and *Pseudomonas aeruginosa* [14,22]. The interactions between these two pathogens have been explored by several studies [14,18, 23,35]. Growing both pathogens together led to increased biofilm production, increased antibiotic tolerance, including to gentamicin and vancomycin, and more severe infections [18,28]. Despite most of the literature focusing on these two pathogens, multiple species are found in chronic wounds [7,13, 14, 36,39], up to 17 genera [40]. One of the three main sources of wound contamination is the surrounding skin, allowing *Staphylococcus*, *Micrococcus* and *Cutibacterium* to infiltrate the wound [36,41,43].

Two common skin commensal bacteria, *Staphylococcus epidermidis* [44,45] and *Micrococcus luteus* [46,48], have both been isolated from surgical wounds and diabetic foot ulcers [36,49] and can be opportunistic pathogens [50,53]. It is suggested that they act as reservoirs of antibiotic resistance genes, which can be transferred to more virulent species [52,54, 55] or display indirect pathogenicity [56,57]. *M. luteus* has also been shown to enhance the infection potential of *S. aureus* [58]. Gram-positive commensal species have also been shown to enhance *P. aeruginosa* virulence [25,59]. Contrarily, *S. epidermidis* produces several effector molecules, including phenol-soluble modulins [60] and an extracellular serine protease [61], which inhibit *S. aureus* growth/biofilm formation. These polymicrobial interactions within wound infections are complicated [9,29, 34, 37] and should be further evaluated, with the eventual aim to help decrease treatment failures [7,9, 56, 62].

Current treatments of chronic wound infections include ciprofloxacin and tobramycin [63,66], among many others [66,67], both broad-spectrum antibiotics. With the increase in antibiotic resistance, there is a limitation of current treatment options. This problem is compounded by biofilm formation [68,70], which is found in wound infections [71,72]. Biofilms provide several benefits to the bacteria; their inclusion within a polymeric matrix offers protection against the environment but, more importantly, prevents the penetration of immune system cells and antimicrobials. This leads to heightened levels of resistance and infections becoming extremely difficult to eradicate, thus leading to the chronicity of the wound [20,70, 73, 74]. The antibiotic resistance of wound-isolated bacteria has also been shown to increase by 800% over 21 days of hospitalization [16], and it has been reported that *P. aeruginosa* and *S. aureus* can delay wound healing [73,75], increasing the duration of hospitalization.

Most of the literature focuses solely on the interactions of *P. aeruginosa* and *S. aureus*, with little information regarding the effect of commensals in such polymicrobial biofilms available. We have recently demonstrated in our group [76] that commensals interact with pathogens, diminishing their virulence and offering protection to eukaryotic cells. The present work explores how the presence of the commensals *S. epidermidis* and *M. luteus* might affect the antibiotic resistance patterns of the pathogens *P. aeruginosa* and *S. aureus*.

## Methods

All absorbance measurements were undertaken on plate readers Infinite F200 or Spark 10M (TECAN, Switzerland). All reagents were obtained from Sigma-Aldrich and used without further purification, unless stated.

### Bacterial strains and growth conditions

The bacterial strains used are listed in Table S1 [76,80]. Bacteria were grown in Lysogeny broth (*P. aeruginosa*) or brain heart infusion (*S. aureus, S. epidermidis* and *M. luteus*) media. For testing in the co-culture model, all four strains were grown in RPMI-1640 without phenol red [supplemented with 10% v/v fetal bovine serum (FBS) and 1% v/v l-glutamine 200 mM]. Appropriate antibiotics (Table S1, available in the online Supplementary Material) were added when needed. All cultures and plates were incubated at 37 °C. Liquid cultures were prepared by inoculating media with single bacterial colonies and incubating at 200 r.p.m.

### Co-culture model

One millilitre of the separate overnight cultures of *S. aureus*, *S. epidermidis*, *M. luteus* and *P. aeruginosa* was pelleted for 1 min at maximum speed (≥12,000 g) and washed with PBS (pH7.4). The cultures were subsequently resuspended in supplemented RPMI-1640 to an OD_600_ 0.1 in a final volume of 5 ml. *S. aureus* and *P. aeruginosa* were further diluted to reach a 1 : 10 (OD_600_ 0.01) and 1 : 100 (OD_600_ 0.001) dilution, respectively. For the polymicrobial studies, the volume required to obtain the above-mentioned ODs for each bacterial species was added together, adjusting the volume of RPMI-1640 accordingly. One hundred microlitres of the normalized cultures were added to each well in a 96-well plate and incubated at 37 °C in a static humid chamber for up to 48 h, with or without treating with antibiotics after the first 24 h of growth, as explained later in this section.

### Bacterial viable counts

After the incubation, cultures were mixed by thoroughly pipetting, and 30 µl was added to 270 µl of PBS and serially diluted down to 10^−8^. Five microlitres was plated in triplicate onto agar containing the appropriate selective antibiotics (Table S1), and viable count was performed after 24 h (48 h for *M. luteus*). Selective plates contained tetracycline for *P. aeruginosa*, erythromycin for *S. aureus*, chloramphenicol for *S. epidermidis* and furazolidone for *M. luteus*. Nalidixic acid combined with colistin was used to inhibit the growth of *P. aeruginosa*. All four strains used had phenotypically distinct colony morphologies, which validated the selective recovery of the four strains.

### Cross-streak assay

Bacterial overnights were washed in PBS and the OD_600_ was normalized to 0.1, 0.01 or 0.001 in supplemented RPMI-1640 for commensals, *S. aureus* and *P. aeruginosa,* respectively, as previously explained. One species was streaked onto agar and allowed to dry, before a second species was streaked perpendicularly, covering all possible combinations. Triplicate sets of plates were streaked out for each species. The plates were incubated for 48 h at 37 °C, before being photographed. The relationship between the two species was scored at 0.25 intervals between 1 (dominant) and −1 (repressed) and then averaged across all triplicates. The dominant score was determined by comparing the growth/survival of colonies of the initial species between the control and dual-species cross-streak. For example, an ~50% reduction in the growth of the initial species gave the dominant species a score of 0.5 and the initial species a score of −0.5. This method was adapted from Pacheco-Moreno *et al*. [81].

### Planktonic MIC

Bacterial strain overnights were washed in PBS, before the OD_600_ of all species was normalized to 0.1 in supplemented RPMI 1640, to allow MIC values to be directly comparable to the wider literature. One hundred microlitres of the bacterial dilutions was added to a 96-well plate, followed by the addition of 100 µl of antibiotic (2× concentration). The plate was then incubated for 24 h in a static humid chamber, before the absorbance (OD_600_) was measured. This data allowed MIC determination. Following this, 30 µl of each well was added to 270 µl of PBS, and 5 µl was plated onto agar containing the appropriate selective antibiotics (Table S1) and incubated overnight. The minimum bactericidal concentration (MBC) was determined through visual inspection for growth. At four pre-selected concentrations, viable counts were performed.

### Minimum Biofilm Eradication Concentration determination

Strains were prepared as explained above and incubated in a static humid chamber for 24 h. Then, 100 µl of antibiotic (2× concentration) was added, and the plate was re-incubated for 24 h. For Minimum Biofilm Eradication Concentration (MBEC) determination, biofilm was disrupted by thoroughly pipetting, and 30 µl of each well was added to 270 µl of PBS. Five microlitres was plated onto agar with selective antibiotics (Table S1) and incubated for 48 h before being visually inspected for growth. At four selected concentrations, viable counts were performed as explained before.

### Figure production

All figures were produced using RStudio [82], which provided a graphical user interface for R programming. The R packages used were ggplot2 [83], ggpubr [84] and corrplot [85].

### Statistical analysis

Mean values of the tested conditions were compared using two-tailed Student’s t-test using the Excel Statistics package XLSTAT (Microsoft Office software), and *P-*values <0.05 were considered statistically significant.

## Results

Previous work from our group [76] highlighted the importance of modelling the ratio of commensals to pathogens during initial skin wound colonization as differences in the relative abundance of species had an impact on antibiotic sensitivity and polymicrobial biofilm formation. Thus, in the present work, those ratios were imitated, being 100 commensals to 10 *S*. *aureus* to 1 *P*. *aeruginosa*, which was achieved by inoculating cultures normalized to an OD_600_ of 0.1 (commensals), 0.01 (*S. aureus*) and 0.001 (*P. aeruginosa*).

### Co-culture caused large changes to bacterial viability in a species-dependent manner

**Fig. 1.**
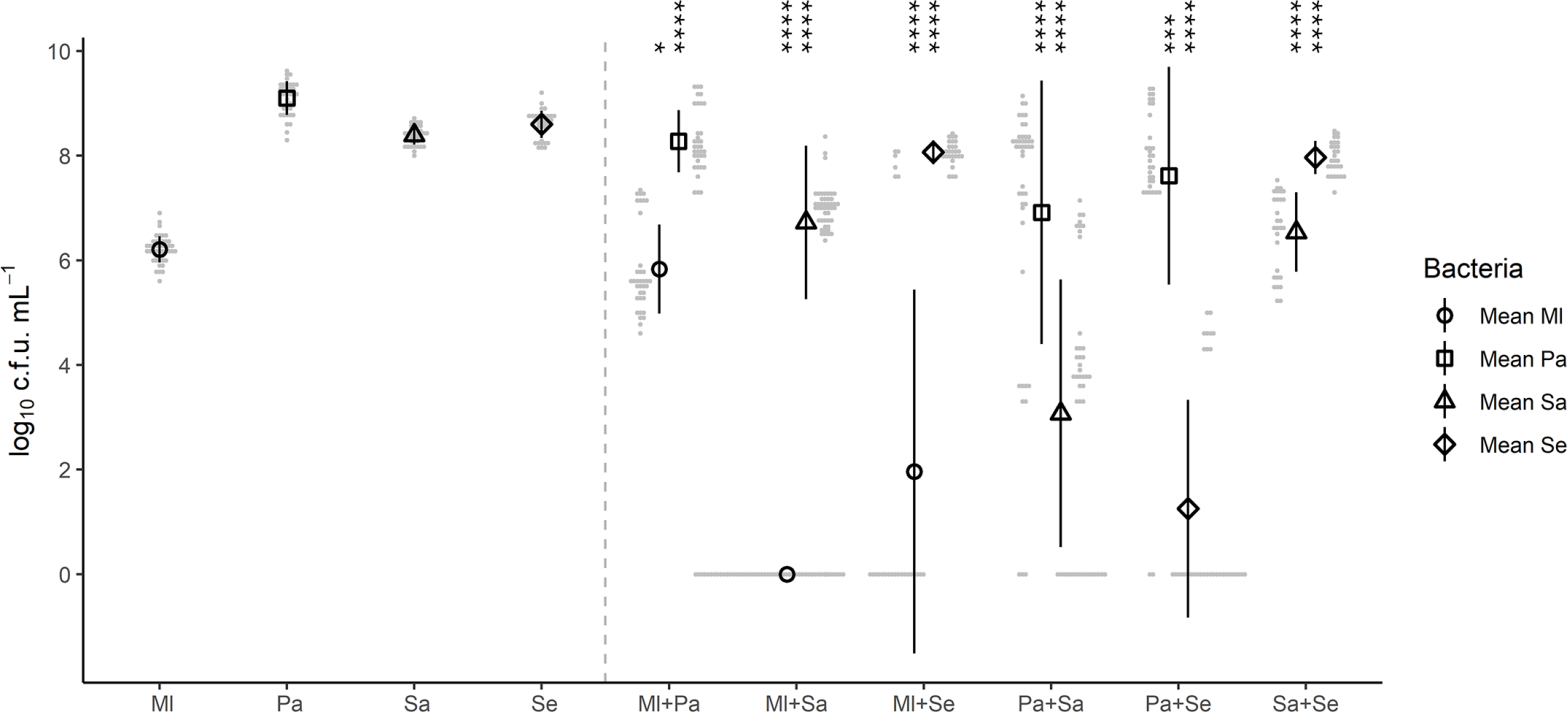
Depending on the combination of species inoculated, the viability of a particular bacterium within the co-culture, after a 48-h incubation, differed. Bacterial species were grown for up to 48 h either alone or as co-culture, in the absence of antibiotics, followed by serially diluting and plating on antibiotic selective media to calculate the viable count (c.f.u.) after a further 24 h of growth. Ml*, M. luteus*; Pa*, P. aeruginosa*; Se*, S. epidermidis*; Sa*, S. aureus*. Nine biological replicates for single species (left-hand side) and six biological replicates for paired (right-hand side) species; comparison of means of the co-culture and the single species was assessed by t-test, with statistical significance displayed as *p<0.05, **p<0.01, ***p<0.001 and ****p<0.0001 (Table S2). Error bars represent sd.

The viability of each bacterium was monitored in a range of mono- and dual-species co-cultures using viable counts. The viability of all species was impacted by co-culturing in comparison to monoculture (Fig. 1, Table S2). The viability of *M. luteus* was impacted by the presence of all three species in co-culture, reducing from 6.3±0.3 log_10_ c.f.u. ml^−1^ to 5.8±0.9 (*p=*0.014) with *P. aeruginosa* to 2.0±3.5 (*p=*4.2×10^−6^) with *S. epidermidis* and to no cell recovery (*p=*1.5×10^−64^) with *S. aureus*. For *P. aeruginosa*, the viable counts were reduced from 9.1±0.3 log_10_ c.f.u. ml^−1^ to 8.3±0.6 (*p=*2.2×10^−9^) with *M. luteus*, to 7.0±2.3 (*p=*6.7×10^−7^) with *S. aureus* and to 7.7±1.9 (*p=*0.0003) with *S. epidermidis*. For *S. aureus*, the viability was reduced from 8.4±0.2 log_10_ c.f.u. ml^−1^ to 6.7±1.5 (*p=*4.1×10^−10^) with *M. luteus*, to 3.1±2.5 (*p=*8.3×10^−18^) with *P. aeruginosa* and to 6.5±0.8 (*p=*7.8×10^−14^) with *S. epidermidis*. Finally, the viability of *S. epidermidis* was reduced from 8.6±0.3 log_10_ c.f.u. ml^−1^ to 8.1±0.2 (*p=*4.8×10^−12^) with *M. luteus*, to 1.3±2.1 (*P=*4.4×10^−20^) with *P. aeruginosa* and to 8.0±0.3 (*p=*9.4×10^−12^) with *S. aureus*. Overall, this highlights that *P. aeruginosa* reduced the viability of both *Staphylococcus* species by 5–6 log_10_ c.f.u. ml^−1^, whilst only reducing its viability by 1–2 log_10_ c.f.u. ml^−1^.

### Interspecies interactions differed between broth co-culture and agar-supported growth

**Fig. 2.**
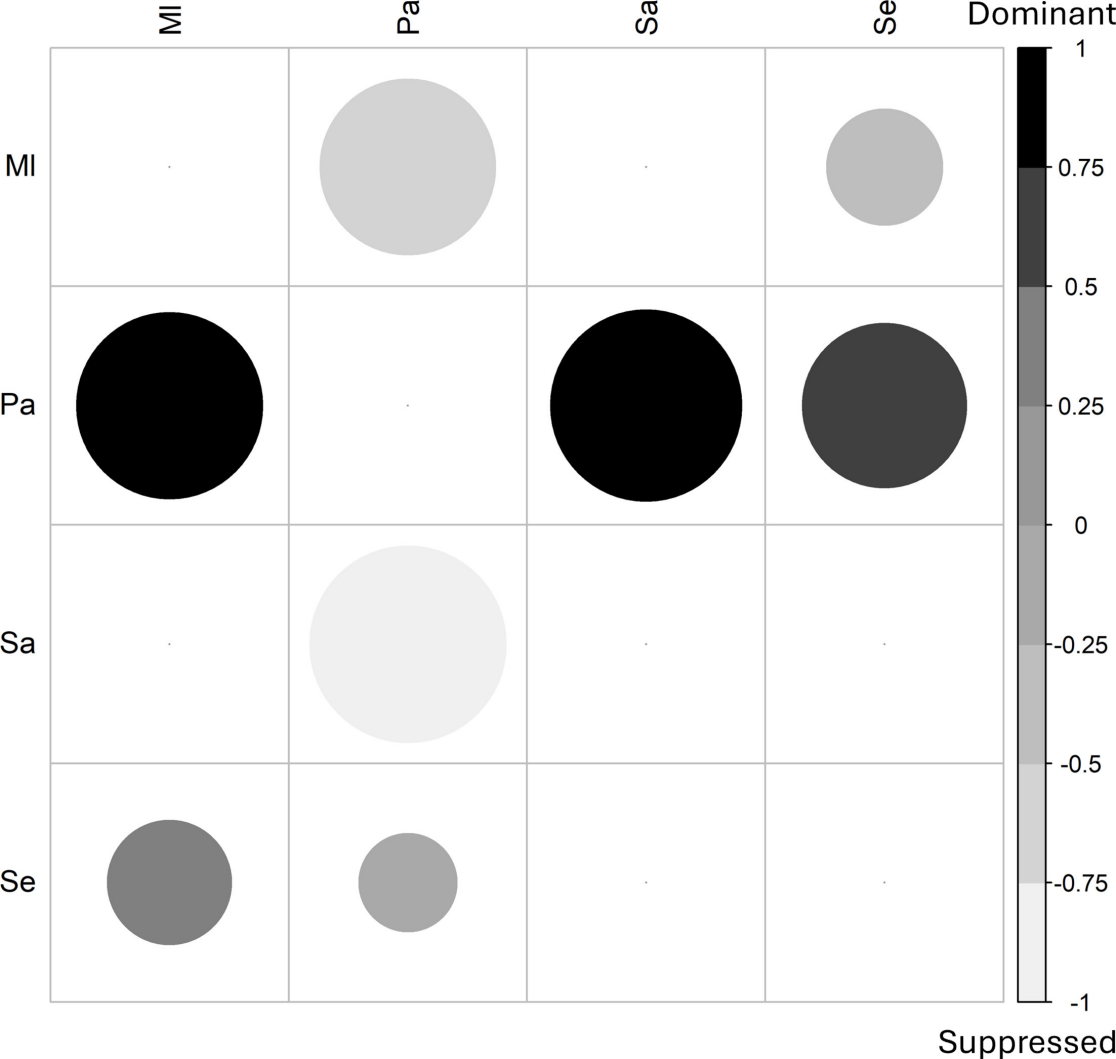
*P. aeruginosa* was dominant over the other three species on agar, whilst the other three species generally co-exist. This matrix summarizes data taken from the cross-streak assay shown in Fig. S1. Species growth was ranked on a scale from 1 (dominant) to −1 (suppressed), based on growth on agar plates. The data was compiled as an average for each pairwise species interaction, performed in triplicate and displayed in the matrix, indicating whether species on the left-hand side were dominant over species at the top of the matrix. The size of the circle indicates the absolute value of the magnitude of the dominance/suppression (smallest circle=0, largest circle is either 1 or −1 depending on colour). Ml*, M. luteus*; Pa*, P. aeruginosa*; Se*, S. epidermidis*; Sa*, S. aureus*. Six biological replicates.

With the aim of gaining corroborative evidence of the interactions taking place between bacterial species suggested by the co-culture viability analysis, a cross-streak assay was performed to evaluate the interactions on agar. This tested whether one species could suppress the growth of a second species (Fig. S1). These results were ranked on a scale of +1 to −1, for dominant to suppressed, for each interaction, and the average scores were displayed as a matrix (Fig. 2).

This data highlighted the dominance of *P. aeruginosa*, indicating growth-inhibitory activity against the other three species of bacteria. *S. aureus* was actively outgrown by *P. aeruginosa* during this on-plate assay, aligning with their known antagonistic relationship [18,28, 29, 31, 86]. In addition, *S. epidermidis* displayed a slight dominance over *M. luteus,* with a score of 0.33±0.29. All other pairwise interactions appeared neutral in nature.

The main differences when comparing the growth in a broth co-culture vs. on agar were related to *M. luteus*. In broth, *M. luteus* could not be recovered from cultures also containing *S. aureus*, whereas no growth inhibition was seen on agar. In contrast, *M. luteus* appeared to be suppressed by *P. aeruginosa* on agar (Fig. S1) but only showed a small decrease in viability from 6.3±0.3 to 5.8±0.9 log_10_ c.f.u. ml^−1^ (*p=*0.014) after co-culture (Fig. 1). The remaining inter-species interactions appeared consistent.

### Antibiotic-mediated eradication was altered by the presence of an additional species

**Fig. 3.**
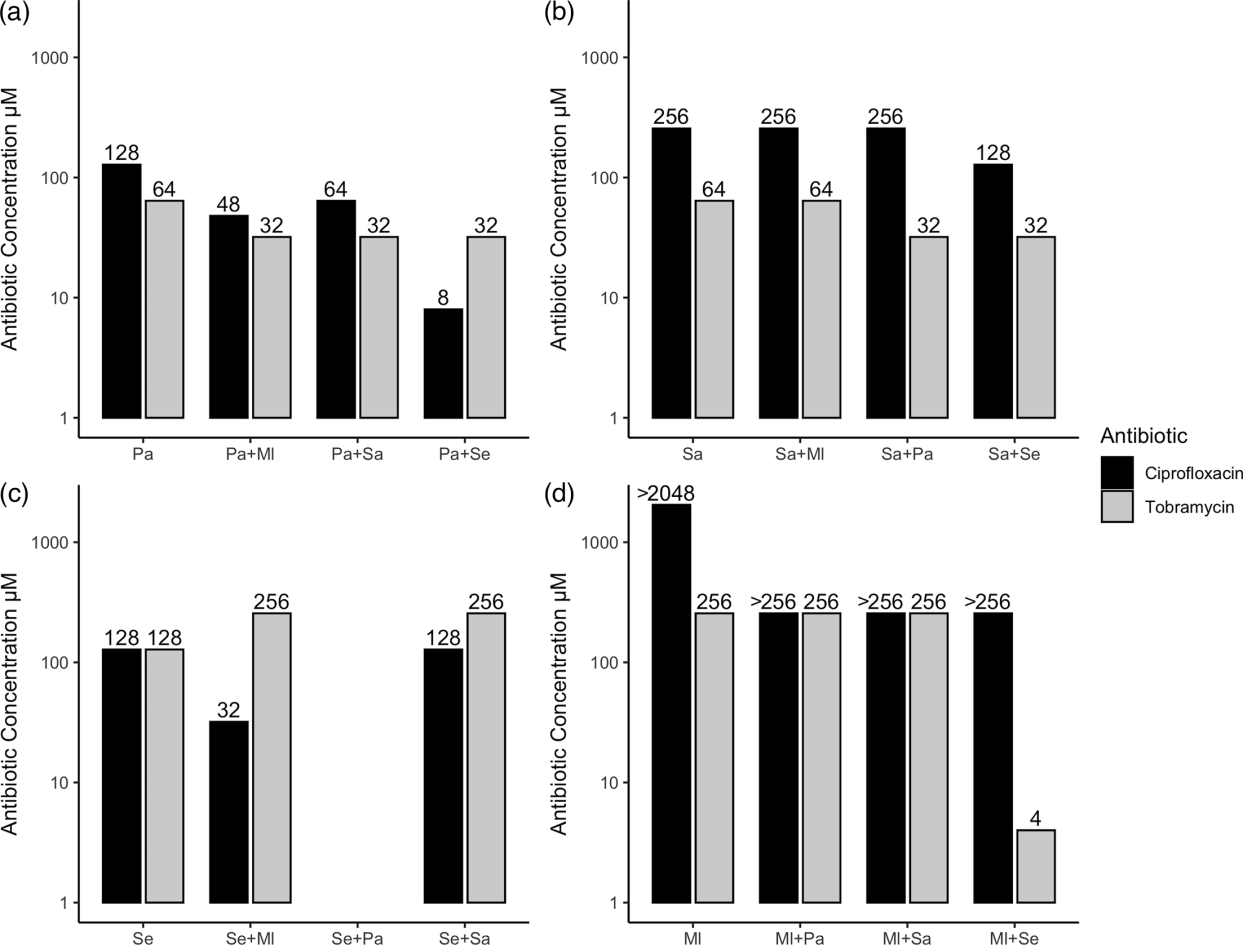
The MBECs for *P. aeruginosa* were consistently lowered when a second bacterial species was present, whereas the antibiotic resistance of the remaining species was only lowered in response to specific species. This figure shows the MBEC values for single- and dual-species cultures following the addition of either ciprofloxacin or tobramycin. The MBEC was determined following 24 h of exposure to antibiotic, by plating onto selective agar and visually inspecting for growth after a further 24 h. (**a**) Pa*, P. aeruginosa*; (**b**) Sa*, S. aureus*; (**c**) Se*, S. epidermidis;* and (d) Ml*, M. luteus*, three biological replicates. No values for MBECs of *S. epidermidis* in the presence of *P. aeruginosa* could be obtained.

Ciprofloxacin and tobramycin were used to explore the effect of an additional species within a co-culture on the antimicrobial resistance, as both antibiotics are commonly used to treat skin wound infections [64,66]. Initially, the MIC and MBC were evaluated as a baseline on the four species separately (Figs S2 and S3). The MBEC was also evaluated on 24-h-old cultures, showing values at least fourfold higher than the MIC/MBC (Figs S2 and S3). This matches with reports that biofilm formation is linked to increased antimicrobial resistance [20,73, 74]. *M. luteus* displayed a high level of resistance to ciprofloxacin, the MIC being 1,024 µM, substantially higher than the 1 µM of *P. aeruginosa* and *S. epidermidis* and 0.25 µM of *S. aureus*. The *M. luteus* MBC/MBEC values to ciprofloxacin were greater than the highest tested concentration of 2,048 µM. In general, all four species were more resistant to ciprofloxacin compared to tobramycin (Fig. 3).

Focussing on ciprofloxacin MBEC values for the dual-species co-cultures (Fig. 3a), *P. aeruginosa* resistance to ciprofloxacin decreased when any other species was present. The greatest reduction in *P. aeruginosa* resistance was in the presence of *S. epidermidis;* however, *S. epidermidis* itself could not be recovered from this condition (Fig. 3c). No values for MBECs of *S. epidermidis* in the presence of *P. aeruginosa* could be obtained. *S. epidermidis* ciprofloxacin resistance was reduced in the presence of *M. luteus* but was unaffected when co-cultured with *S. aureus. M. luteus* MBEC values remained greater than the ciprofloxacin concentration tested, so any effects on it by other species could not be determined (Fig. 3d). For *S. aureus*, MBEC values remained unchanged, apart from a twofold decrease in MBEC when paired with *S. epidermidis* (Fig. 3b).

For tobramycin MBECs, the same trend of resistance reduction when any other species was present was also observed for *P. aeruginosa* (Fig. 3a)*.* Conversely, *S. epidermidis* showed an increased resistance when grown with other species, except when it was eradicated by *P. aeruginosa* (Fig. 3c). *M. luteus* resistance was not altered except when grown with *S. epidermidis*, when it showed a large reduction in its resistance (Fig. 3d). Finally, *S. aureus* also showed a decreased resistance (twofold) when paired with either *S. epidermidis* or *P. aeruginosa* (Fig. 3b).

To evaluate the effect of sub-inhibitory doses of antibiotic, viable counts were undertaken at three antibiotic concentrations and in the absence of antibiotic treatment. The concentrations selected for ciprofloxacin were twofold higher than tobramycin, due to the increased resistance of *M. luteus*, *P. aeruginosa* and *S. aureus* to ciprofloxacin.

### *P. aeruginosa* tobramycin resistance was reduced in co-culture

**Fig. 4.**
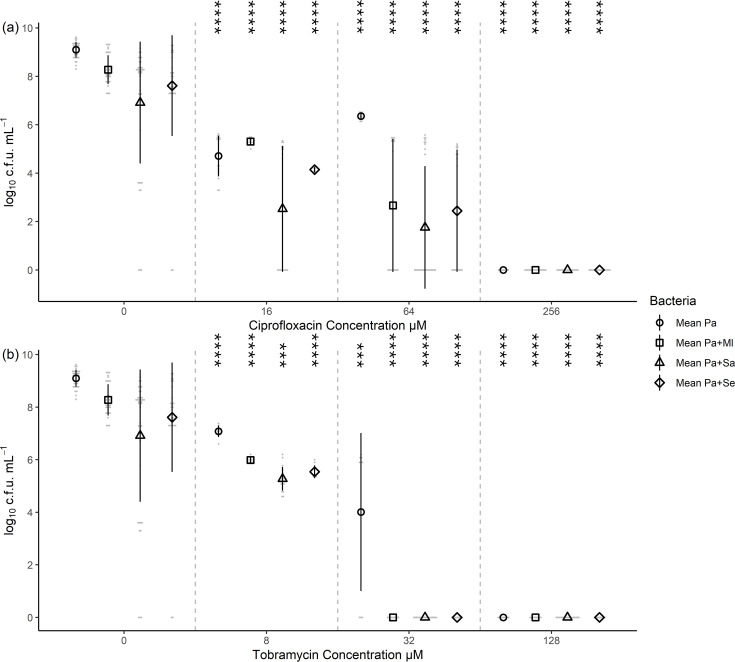
*P. aeruginosa* viable count decreased in co-culture. This change was more pronounced with the bactericidal antibiotic tobramycin compared to the bacteriostatic antibiotic ciprofloxacin. This figure indicates the viable counts of *P. aeruginosa* following single- and dual-species culture exposure to antibiotic for 24 h. (**a**) Exposure to ciprofloxacin. (**b**) Exposure to tobramycin. Comparison of means of the co-cultures with the single-species growth was assessed by t-test, with statistical significance displayed as *p<0.05, **p<0.01, ***p<0.001 and ****p<0.0001 for all studied pairs (Table S3). Ml*, M. luteus*; Pa*, P. aeruginosa*; Se*, S. epidermidis*; Sa*, S. aureus*. Six biological replicates for zero antibiotic and three biological replicates for the plus antibiotic conditions. Error bars represent sd.

As previously shown (Fig. 1), co-culture reduced the viability of *P. aeruginosa* by at least one log. During co-culture, the addition of 64 µM ciprofloxacin reduced *P. aeruginosa* viable count from 6.4±0.2 log_10_ c.f.u. ml^−1^ to 2.7±2.8 (*p=*2.6×10^−5^) with *M. luteus*, to 1.8±2.5 (*p=*6.5×10^−10^) with *S. aureus* and to 2.4±2.5 (*p=*4.5×10^−6^) with *S. epidermidis*. This had no effect on the antibiotic resistance, as *P. aeruginosa* could be recovered at 64 µM ciprofloxacin with or without other species present (Fig. 4a, Table S3). However, the *P. aeruginosa* resistance to tobramycin was reduced below 32 µM in the presence of any other species, as no viable bacteria could be recovered (*p=*0.004), from 4.0±3.0 log_10_ c.f.u. ml^−1^ in monoculture with 32 µM tobramycin (Fig. 4b, Table S3). The reduction in viability was also observed with 8 µM tobramycin, from 7.1±0.2 log_10_ c.f.u. ml^−1^ to 6.0±0.1 (*p=*2.6×10^−8^) with *M. luteus*, to 5.3±0.5 (*p=*2.6×10^−13^) with *S. aureus* and to 5.5±0.2 (*p=*2.4×10^−10^) with *S. epidermidis*.

### *S. aureus* tobramycin resistance was also reduced in co-culture, although in a species-dependent manner

**Fig. 5.**
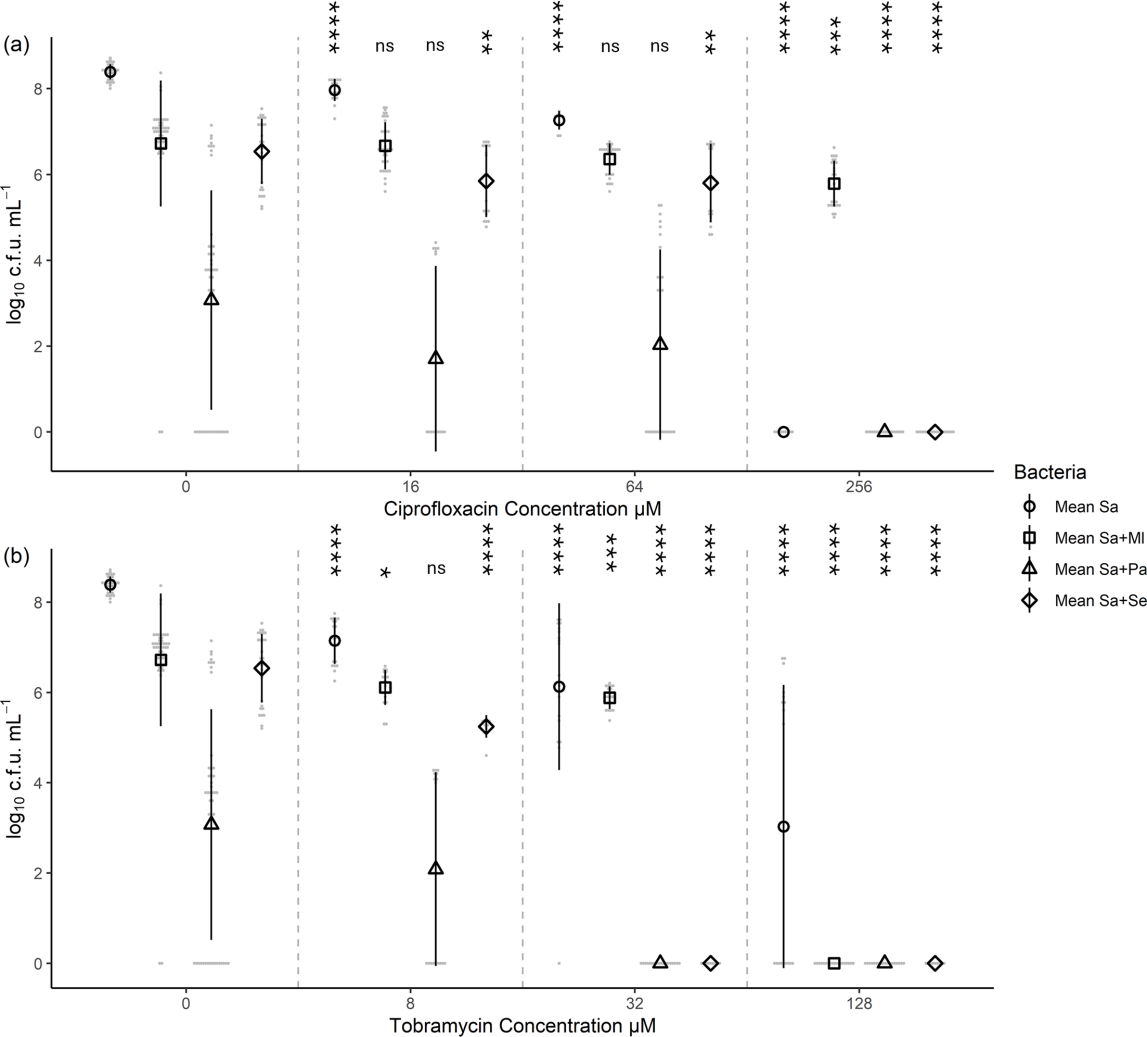
*S. aureus* viable count decreased in co-cultures. This change was more pronounced with the bactericidal antibiotic tobramycin compared to the bacteriostatic antibiotic ciprofloxacin. This figure indicates the viable counts of *S. aureus* following single- and dual-species culture exposure to antibiotic for 24 h. (a) Exposure to ciprofloxacin. (b) Exposure to tobramycin. Comparison of means for the co-cultures with the single-species growth was assessed by t-test, with statistical significance displayed as *p<0.05, **p<0.01, ***p<0.001 and ****p<0.0001 (Table S4). Ml, *M. luteus*; Pa, *P. aeruginosa*; Se = *S. epidermidis*; Sa, *S. aureus*. Six biological replicates for zero antibiotic and three biological replicates for the plus antibiotic conditions. Error bars represent sd.

Similar trends were found for *S. aureus*. The addition of 64 µM ciprofloxacin reduced the viable count from 7.3±0.2 log_10_ c.f.u. ml^−1^ to 6.4±0.4 (*p=*4.1×10^−9^) with *M. luteus*, to 2.0±2.2 (*p=*1.7×10^−12^) with *P. aeruginosa* and to 5.8±0.9 (*p=*2.6×10^−6^) with *S. epidermidis* (Fig. 5a, Table S4). The antibiotic resistance was unaffected, except the addition of *M. luteus* increased the ciprofloxacin resistance of *S. aureus* up to 256 µM, with 5.8±0.5 log_10_ c.f.u. ml^−1^ being recovered.

For the tobramycin-treated co-cultures, the addition of *S. epidermidis* or *P. aeruginosa* at 32 µM tobramycin eliminated *S. aureus* (*p=*8.5×10^−11^)*,* but tobramycin resistance was still at least 8 µM. Whilst the addition of *M. luteus* had no significant effect on the viable count, being 6.1±1.9 log_10_ c.f.u. ml^−1^ in monoculture compared to 5.9±0.3 (*p=*0.58) with *M. luteus* at 32 µM tobramycin (Fig. 5b, Table S4). However, the addition of any second species resulted in no recovery of *S. aureus* at 128 µM tobramycin, compared to treating the monoculture (*p=*0.0008).

### The commensals *S. epidermidis* and *M. luteus* had opposite outcomes when co-cultured with *P. aeruginosa*

**Fig. 6.**
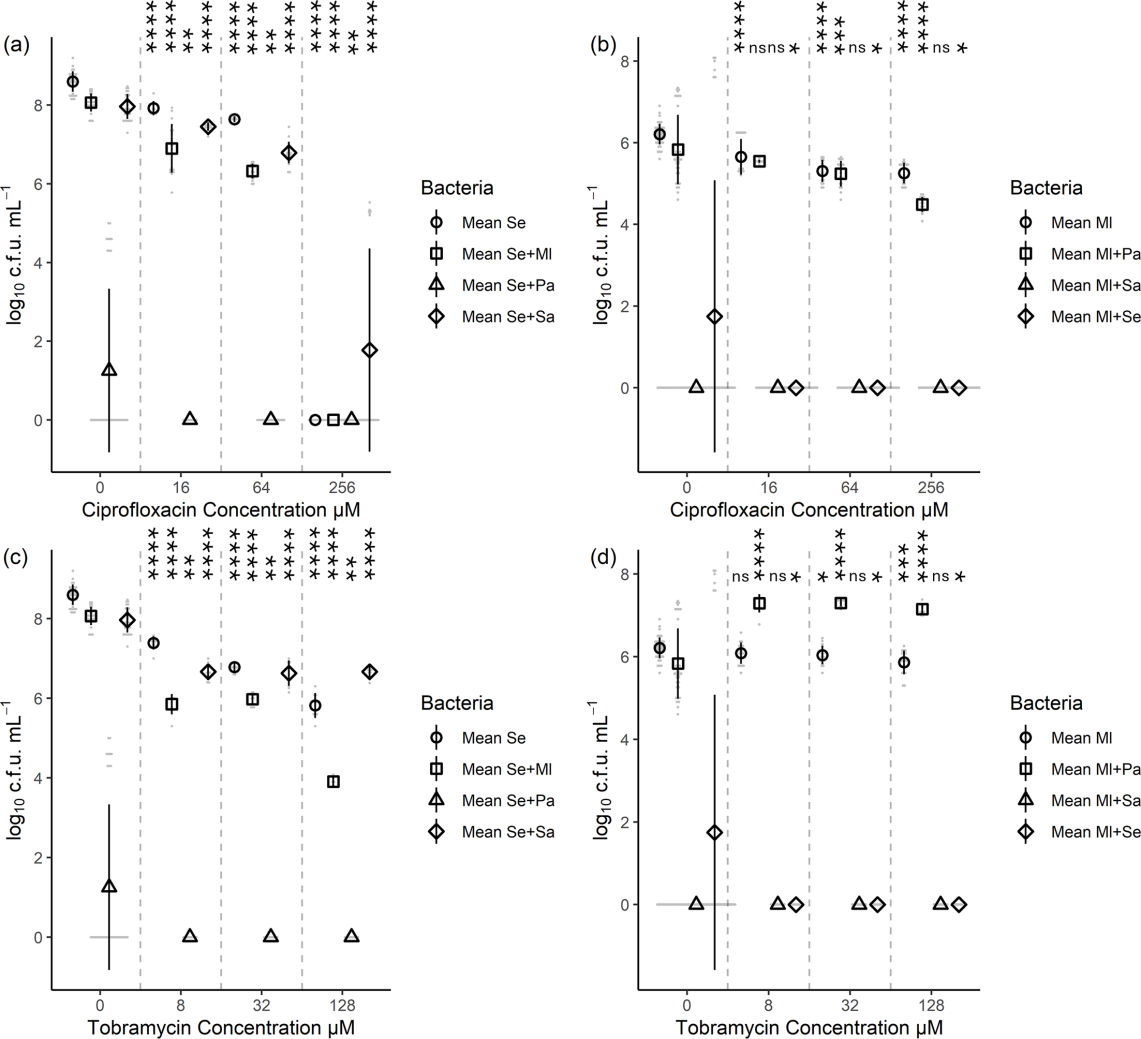
*S. epidermidis* viability is reduced by co-culture and addition of antibiotics, with the largest decreases in viability occurring with *P. aeruginosa* co-culture. In contrast, *M. luteus* showed equivalent or increased resistance when co-cultured with *P. aeruginosa*, with limited recovery in the presence of either *Staphylococcus* species. This figure indicates the viable counts following culture exposure (single and dual-species) to ciprofloxacin or tobramycin for 24 h, with each graph indicating the viable counts for a commensal and antibiotic. (a) *S. epidermidis* + ciprofloxacin, (b) *M. luteus* + ciprofloxacin, (c) *S. epidermidis* + tobramycin and (d) *M. luteus* + tobramycin. Comparison of means of the co-cultures with the single-species growth was assessed by t-test, with statistical significance displayed as *p<0.05, **p<0.01, ***p<0.001 and ****p<0.0001 for all studied pairs (Table S5). Ml, *M. luteus*; Pa, *P. aeruginosa*; Se, *S. epidermidis*; Sa, *S. aureus*. Six biological replicates for zero antibiotic and three biological replicates for the plus antibiotic conditions. Error bars represent sd.

Co-culturing *S. epidermidis* with *P. aeruginosa* translated into complete eradication of the former when treated with either ciprofloxacin or tobramycin, as it could not be recovered from any of the antibiotic concentrations tested, whilst monoculture *S. epidermidis* grew up to 64 µM of ciprofloxacin (*p=*1.0×10^−17^) and up to 128 µM of tobramycin (*p=*1.2×10^−11^). Regarding the other micro-organisms, commensal *M. luteus* had a small but significant effect on *S. epidermidis*. For 64 µM ciprofloxacin, a reduction from 7.6±0.1 log_10_ c.f.u. ml^−1^ to 6.3±0.2 (*p=*2.8×10^−19^) in viability. For 128 µM tobramycin treatment, the reduction in viable count was from 5.8±0.3 log_10_ c.f.u. ml^−1^ to 3.9±0.1 (*p=*4.0×10^−9^). However, co-culture with *S. aureus* increased *S. epidermidis* resistance to ciprofloxacin from 64 to 256 µM, recovering 1.7±2.6 log_10_ c.f.u. ml^−1^ (*p=*0.0097) (Fig. 6a, c, Table S5).

In contrast, when co-cultured with *P. aeruginosa*, *M. luteus* could be recovered from any of the antibiotic concentrations tested. There was no significant difference in viable counts at 16 or 64 µM ciprofloxacin, but at 256 µM ciprofloxacin, the viability decreased from 5.3±0.3 log_10_ c.f.u. ml^−1^ to 4.5±0.2 (*p=*1.0×10^−11^) (Fig. 6b, Table S5). The viability in tobramycin was on average one log higher than the monoculture; for example, at 32 µM tobramycin, the viable counts increased from 6.0±0.2 log_10_ c.f.u. ml^−1^ to 7.3±0.1 (*p=*6.3×10^−17^) (Fig. 6d, Table S5). In the presence of either antibiotic, *M. luteus* recovery from co-cultures with either *Staphylococcus* species was not possible (*P*-values between 8.9×10^−19^ and 6.2×10^−26^, Table S5). Viability was thus assumed to be zero in these conditions.

## Discussion

The first step in this study was to establish a robust and reliable co-culture of the chosen commensals and pathogens that would be realistic and amenable to the downstream analysis of antimicrobials. The ratio of commensals to pathogens in an infected wound is unknown; however, it was hypothesized that the number of pathogenic cells would be lower than the number of commensal bacteria. The microbiota range is from 10^2^ to 10^6^ c.f.u. cm^−2^ on healthy skin [87], and the infecting dose of *P. aeruginosa* for wounded skin has been reported to be ~10^3^ c.f.u. and less than 10^5^ c.f.u. for *S. aureus* when the infection route was topical [88]. Previous work in our group showed that different ratios of commensals to pathogens had an impact on the polymicrobial biofilms that formed [76]. A higher initial concentration of commensals, to approximate skin microbiome bacterial loads, allowed them to better colonize a keratinocyte layer and offer protection against pathogens [76]. In this study, we used the ratio of 100 commensals (*S. epidermidis* or *M. luteus*) to 10 *S. aureus* to 1 *P. aeruginosa,* to explore the impact of different combinations of species on susceptibility to two antibiotics used in the treatment of wounds: bactericidal tobramycin and bacteriostatic ciprofloxacin. The supplementation of the growth medium with FBS is unlikely to affect the antibiotic concentration, due to these antibiotics’ low protein binding in serum [89,90]. To allow viable counts for each individual species, we selected strains containing plasmids with antibiotic resistance genes (Table S1). Thus, *P. aeruginosa* was selected using tetracycline, *S. aureus* was selected using erythromycin, *S. epidermidis* was selected using chloranphenicol, and *M. luteus* was selected using furazolidone. Additionally, antibiotics nalidixic acid and colistin were used to inhibit the growth of *P. aeruginosa*.

In a polymicrobial community, different species impact each other via direct or indirect competition and synergism or have no interaction [14,37, 91]. It follows that this influences bacterial viability and physiology, which in turn could alter susceptibility to antibiotic treatment. In addition, the recovery of viable cells from biofilms may differ between each species due to their different biofilm matrices [92,93]; however, as we are comparing each species’ viable counts within the species, this should limit the impact on our findings.

The viability of all four bacterial species included in this study was at least tenfold lower when grown in co-culture compared to monoculture (Fig. 1). Most notably, *M. luteus* viability was undetectable when it was co-cultured with *S. aureus* (*p=*1.5×10^−64^); however, the nature of the underlying mechanism of this is not clear. *P. aeruginosa* is known to produce a number of factors which could negatively affect the growth of other species, including pyocyanin, 4-hydroxy-2-heptylquinoline N-oxide and the LasA protease [25,29, 91, 94]. Together with competition for resources [87,95], this could explain the large decreases in viability of the other species (Fig. 1) and its dominance in the cross-streak growth assay on agar (Figs 2 and S1). Viability of the other three species decreased by 5.3±2.7 log_10_ c.f.u. ml^−1^ (*p=*8.3×10^−18^) for *S. aureus,* by 7.3±2.4 (*p=*4.4×10^−20^) for *S. epidermidis,* and by 0.5±1.2 (*p=*0.014) for *M. luteus* in co-culture with *P. aeruginosa*. Our use of manual pipetting for disruption may have influenced the absolute viable counts obtained, and any future studies should employ a more standardized disruption method such as sonication.

As expected, not all the species influenced each other similarly on agar and in broth co-culture. Spatially structured environments, such as agar, influence behaviour/selection differently compared to spatially unstructured environments, such as broth [96]. This leads to differences in compound dispersal and cell–cell interactions/contact in liquid vs. solid media [97,98]. The reduction in virulent toxin production by *P. aeruginosa* in unstructured environments [96] could explain the small reduction in viability of 0.5±1.2 (*p=*0.014) for *M. luteus* during co-culture in broth, compared to strong suppression of *M. luteus* on agar. In contrast, *S. aureus* can produce several *M. luteus* inhibiting bacteriocins [99,101], whose broader diffusion in broth compared to agar could cause greater growth inhibition. The slightly antagonistic relationship between *S. epidermidis* and *M. luteus* could be associated with the different distributions of these commensals on the skin [37,52, 102]. Limitations of species recovery/analysis have been considered below, with a limit of detection of 1×10^3^ for standard viable counts and 1×10^5^ for viable counts of wells containing antibiotics.

Evaluation of the resistance of single vs. dual-species cultures to the antibiotics ciprofloxacin and tobramycin revealed that, in general, all four species were more resistant to the fluoroquinolone ciprofloxacin (Fig. 3a). This could be attributed to its bacteriostatic activity at lower concentrations [103,104], compared to the solely bactericidal activity of the aminoglycoside tobramycin [105]. The bacteriostatic activity of ciprofloxacin can also be responsible for the discrepancies encountered between MBECs and viable counts, as viable bacteria were recovered from the previously stated MBEC. This could be explained by the methodology adapted from Cruz *et al.* and Thieme *et al.* [106,107], as onefold dilution of the culture was seeded to visually determine growth, thus carrying some residual bacteriostatic antibiotic, but the greater number of serial dilutions performed for the viable count was able to wash out antibiotic traces. The use of repeat pipette disruption of the biofilm in our study, compared to sonication-based disruption, could have also impacted the viable counts. Sonication has more recently been shown to more robustly break up biofilm aggregates for viable counts [108,109].

*P. aeruginosa* showed a greater reduction in antibiotic resistance when grown in a co-culture with *S. epidermidis* for both MBECs, from 128 to 8 µM ciprofloxacin and 64 to 32 µM tobramycin (Fig. 3). The *P. aeruginosa* viable counts also significantly reduced from 6.4±0.2 log_10_ c.f.u. ml^−1^ to 2.4±2.5 (*p=*4.5×10^−6^) in 64 µM ciprofloxacin and from 4.0±3.0 log_10_ c.f.u. ml^−1^ to no recoverable cells (*p=*0.004) in 32 µM tobramycin (Fig. 4). *P. aeruginosa* has been shown to alter gene expression and increase its virulence in response to other bacterial species, especially Gram-positive bacteria [25,59, 95, 110, 111]. This increased virulence and killing may be at the expense of biofilm formation during the first 24 h and hence lead to reduced antibiotic resistance in the second 24 h. On the other hand, Gram-positive quorum sensing molecules [autoinducer-2 (AI-2)] [59,91, 110] and their peptidoglycan [7,25] have been shown to influence *P. aeruginosa* behaviour through modulation of distinct but overlapping subsets of genes [59]. These signalling systems can integrate [91] into the already complex quorum sensing systems of *P. aeruginosa*, including acyl homoserine lactones (AHLs) and *Pseudomonas* quinolone signal (PQS)-based signalling [112,113], and alter its responses. As *P. aeruginosa* was added at low initial bacterial dose, the ratio of AHLs/PQS compared to AI-2 may influence the relative activation of biofilm formation vs. virulence factor production.

In the case of *S. aureus,* the addition of *S. epidermidis* reduced the MBEC values twofold for both ciprofloxacin and tobramycin (Fig. 3). The maximum reduction in viable counts was observed when 32 µM tobramycin-treated *S. aureus* was co-cultured with either *S. epidermidis* or *P. aeruginosa,* reducing from 6.1±1.9 log_10_ c.f.u. ml^−1^ to no recoverable cells (*p=*8.5×10^−11^) (Fig. 5). However, the addition of *M. luteus* increased *S. aureus* viable counts during 256 µM ciprofloxacin treatment from no recoverable cells to 5.8±0.5 log_10_ c.f.u. ml^−1^ (*p=*1.0×10^−28^). The antagonism between *S. aureus* and *P. aeruginosa* has been widely documented [29,31, 91, 94, 95, 114]. However, during planktonic growth with *P. aeruginosa*, the tobramycin tolerance of *S. aureus* had been shown to increase, due to small-colony variant selection [24,28]. This may highlight a distinction in their interactions between planktonic vs. biofilm growth and the state of the cells during antibiotic treatment. Regarding the antagonism with *S. epidermidis*, this bacterium is known to produce molecules that can inhibit the quorum sensing *agr* system from *S. aureus* [115,116] or produce an extracellular serine protease, which has been shown to destroy *S. aureus* biofilm formation [61,117], thus translating into decreased antibiotic resistance. The potential positive interactions of *M. luteus* on *S. aureus* have been documented in mouse infection models, where *M. luteus* presence enhanced *S. aureus* virulence and survival, despite *M. luteus* being cleared by the immune system [58,118]. This mirrors the results found in the current study. *M. luteus* could not be recovered following co-culture (Figs 1 and 6) but did affect *Staphylococcus* antibiotic resistance. *Staphylococcus* species have also been shown to gain positive benefits from other species without affecting these species in return [119]. However, as the resistance mechanism of *M. luteus* to ciprofloxacin is unknown, we cannot rule out degradation by this commensal, leading to perceived increased *S. aureus* resistance.

*S. epidermidis* antibiotic resistance trends were similar to *S. aureus*. The noticeable exception is the complete eradication by *P. aeruginosa* and hence lack of MBEC values or viable counts data. *P. aeruginosa* has been shown to disperse *S. epidermidis* biofilms and then lyse the planktonic cells after 18 h of co-culture, preventing co-infections of skin wounds [120,121]. The obtained results are less cohesive for co-culture with the other two species. When co-cultured with *S. aureus, S. epidermidis* only showed a small viable count reduction from 8.6±0.3 log_10_ c.f.u. ml^−1^ to 8.0±0.3 (*p=*9.4×10^−12^) but maintained or increased MBEC values. The interactions of *M. luteus* and *S. epidermidis* were more divergent, with a twofold increase in the MBEC of tobramycin but a fourfold decrease of the ciprofloxacin MBEC (Fig. 3c). These results could indicate a more complex interaction network between these two commensal species, which might have a link to their different distribution on the skin [37,52, 102]. Alternatively, indirect resource competition [91] may have caused the decrease in viability at 64 µM ciprofloxacin from 7.6±0.1 log_10_ c.f.u. ml^−1^ to 6.3±0.2 (*p=*2.8×10^−19^) and ciprofloxacin resistance. *M. luteus* appears to be inherently resistant to ciprofloxacin which would impart it with unimpaired growth [122,126], allowing it to indirectly outcompete *S. epidermidis* and decrease its antibiotic resistance/survival. The increase in tolerance to tobramycin may indicate an induced resistance mechanism.

Finally, the MBEC values obtained for *M. luteus* following ciprofloxacin treatment implied that this species may be inherently resistant (Fig. 3d), although a wide range of MIC for this bacterium has been previously described, ranging from 0.5 µg ml^−1^ to resistant [122,126]. The viable counts obtained showed a significant decrease following ciprofloxacin addition but remained within a one log_10_ decrease, from 6.2±0.3 log_10_ c.f.u. ml^−1^ to 5.7±0.4 (*p=*5×10^−5^), 5.3±0.3 (*p=*6.3×10^−13^) and 5.3±0.3 (*p=*1.3×10^−14^) for 16, 64 and 256 µM ciprofloxacin, respectively (Fig. 6b). The decrease in tobramycin MBEC from 256 to 4 µM when *M. luteus* was grown with *S. epidermidis*, alongside *S. epidermidis* increased tobramycin MBEC from 128 to 256 µM during co-culture, could suggest uncharacterized interactions are occurring between these two commensal species (Fig. 3). This could also be linked to reduced or no viability of *M. luteus* following 48 h of co-culture with either *Staphylococcus* species. Whilst the MBEC following tobramycin treatment of *M. luteus* with *P. aeruginosa* was unchanged, the viable counts increased from an average of 6.0±0.3 log_10_ c.f.u. ml^−1^ to 7.2±0.2 (*p=*4.7×10^−11^) across all three tobramycin concentrations compared to *M. luteus* alone. This occurred even when no *P. aeruginosa* could be recovered at certain tobramycin concentrations and could imply a priming effect by this species to increase tobramycin tolerance of *M. luteus*.

These results highlight several areas for future exploration. Particularly *M. luteus*, it is a relatively common skin commensal bacteria and opportunistic pathogen, which displayed higher levels of resistance compared to pathogenic species, whilst also growing in the presence of *P. aeruginosa.* However, given the limited recovery of *M. luteus* during co-culture, our strain’s resistance profile is unlikely to have impacted the wider findings of this co-culture study. Another avenue of future research could be elucidating the mechanisms behind the effect of co-culture environments on the antibiotic resistance of *P. aeruginosa* and *S. aureus*, as it could help in the fight against antimicrobial resistance. These interspecies effects could be investigated through transcriptomic analysis during co-culture or monitoring levels of known antagonistic molecules. Future iterations of this model could benefit from the inclusion of clinical strains or the addition of a host skin boundary/wound environment to improve its clinical applicability [76].

In conclusion, this work showed that ciprofloxacin and tobramycin resistance was altered in bacterial co-cultures in a composition-dependent manner. Given that growth within biofilms causes heightened drug resistance, quantified by MBECs being >100× higher than associated MICs, models like the one described are needed to assess biofilm drug efficacy during development. Our work also showed a decrease in *P. aeruginosa* viability when it was grown with another bacterial species, despite its growth inhibitory activity over these other species. It is highlighted that commensal species had a significant impact on the antibiotic resistance and viable counts of pathogenic species involved in skin wound infections. This underscores the importance of including commensal species and the use of more advanced *in vitro* systems to model species–species interactions.

## Supplementary material

10.1099/jmm.0.002126 Uncited Fig. S1.

## Abbreviations

AHL: acyl homoserine lactone
AI-2: autoinducer-2
FBS: fetal bovine serum
MBC: minimum bactericidal concentration
MBEC: minimum biofilm eradication concentration
PQS: *Pseudomonas* quinolone signal
