# Supplementary material for: Can commensals alter pathogen’s antibiotic resistance during co-culture?
Source: J Med Microbiol. 2026 Feb 24;75(2):002126. doi: 10.1099/jmm.0.002126 (PMC12931886; doi:10.1099/jmm.0.002126)

Supplementary material

Can commensals alter pathogen's antibiotic resistance within biofilms?

Supplementary table 1: Bacterial Strains and antibiotics used in this study.

Microorganism	Strain	Plasmids	Antibiotic Resistance	Source/ Reference:
Micrococcus luteus	2665		Furazolidone, Nalidixic acid & Colistin (all 10 μg ml^{-1})	Rokem *et al.* 2011 (76), Jordana-Lluch *et al.* 2020 (49)
Pseudomonas aeruginosa	PA01-Nottingham	pME6032 -mCherry	Tetracycline (125 μg ml^{-1})	Ortori *et al.* 2011 (77)
Staphylococcus aureus	SH1000		Nalidixic acid & Colistin (all 10 μg ml^{-1})	O'Neill *et al.* 2010 (78)
Staphylococcus aureus	SH1000	pmKAT	Erythromycin (20 μg ml^{-1}), Nalidixic acid & Colistin (all 10 μg ml^{-1})	Jordana-Lluch *et al.* 2020 (49)
Staphylococcus epidermidis	1457		Nalidixic acid & Colistin (all 10 μg ml^{-1})	Galac *et al.* 2017 (79)
Staphylococcus epidermidis	1457	pSB2019 -gfp	Chloramphenicol, Nalidixic acid & Colistin (all 10 μg ml^{-1})	Jordana-Lluch *et al.* 2020 (49)

<u>Supplementary Table 2</u>: Viable Count Data of Single and Dual-Species Biofilms (Figure 1). Biofilm was prepared as described in methods. Briefly, PBS washed overnight cultures were normalised to 0.1 (commensals), 0.01 (*S. aureus*) and 0.001 (*P. aeruginosa*). After 48h, viable count was performed. Excel statistics package was used to perform a t-Student comparing means among different conditions. A *p*-value ≤ 0.05 was considered statistically significant. **Ml:** *M. luteus;* **Pa***: P. aeruginosa;* **Sa:** *S. aureus;* **Se:** *S. epidermidis*

**Species**	**Viable Count Mean (log_{10}CFUs/mL)**					***p*-values**			
	**Single Species**	**+ Ml**	**+ Pa**	**+ Sa**	**+ Se**	**+ Ml**	**+ Pa**	**+ Sa**	**+ Se**
**Ml**	6.3±0.3	N/A	5.8±0.9	0.0	2.0±3.5	N/A	0.014	$1.5x10^{-64}$	$4.2x10^{-6}$
**Pa**	9.1±0.3	8.3±0.6	N/A	7.0±2.3	7.7±1.9	$2.2x10^{-9}$	N/A	$6.7x10^{-7}$	$2.8x10^{-4}$
**Sa**	8.4±0.2	6.7±1.5	3.1±2.5	N/A	6.5±0.8	$4.1x10^{-10}$	$8.3x10^{-18}$	N/A	$7.8x10^{-14}$
**Se**	8.6±0.3	8.1±0.2	1.3±2.1	8.0±0.3	N/A	$4.8x10^{-12}$	$4.4x10^{-20}$	$9.4x10^{-12}$	N/A

<u>Supplementary Table 3</u>: *P. aeruginosa* Viable Count Averages in single- and dual-species biofilm following antibiotic addition. Data from Figure 4. Biofilm was prepared as described in methods. Briefly, PBS washed ON cultures were normalised to 0.1 (commensals), =0.01 (S. aureus) and =0.001 (P. aeruginosa). After 24h, antibiotic (ciprofloxacin or tobramycin) was added at the desired concentrations and biofilms were further incubated 24h. After that time, viable count was performed. Excel statistics package was used to perform a t-Student comparing means among different conditions. A p-value ≤ 0.05 was considered statistically significant. **Ml**: *M. luteus*; **Pa**: *P. aeruginosa*; **Sa**: *S. aureus*; **Se:** *S. epidermidis*

**Species**	**Viable Count Mean (log_{10}CFUs/mL)**				***p*-values**		
**Ciprofloxacin Addition (μM)**	**0**	**16**	**64**	**256**	**16**	**64**	**256**
Pa	9.1±0.3	4.7±0.8	6.4±0.2	0.0	$5.0x10^{-15}$	$2.2x10^{-25}$	$1.7x10^{-52}$
Pa + Ml	8.3±0.6	5.3±0.2	2.7±2.7	0.0	$1.1x10^{-27}$	$1.0x10^{-7}$	$1.0x10^{-40}$
Pa + Sa	6.9±2.5	2.5±2.6	1.8±2.5	0.0	$9.8x10^{-7}$	$2.2x10^{-11}$	$2.6x10^{-22}$
Pa + Se	7.6±2.1	4.1±0.1	2.4±2.5	0.0	$5.9x10^{-11}$	$2.9x10^{-8}$	$2.7x10^{-20}$
**Tobramycin Addition (μM)**	**0**	**8**	**32**	**128**	**8**	**32**	**128**
Pa	9.1±0.3	7.1±0.2	4.0±3.0	0.0	$2.1x10^{-14}$	$9.5x10^{-4}$	$1.7x10^{-52}$
Pa + Ml	8.3±0.6	6.0±0.1	0.0	0.0	$1.2x10^{-23}$	$1.0x10^{-40}$	$1.0x10^{-40}$
Pa + Sa	6.9±2.5	5.3±0.5	0.0	0.0	$1.1x10^{-4}$	$2.6x10^{-22}$	$2.6x10^{-22}$
Pa + Se	7.6±2.1	5.5±0.2	0.0	0.0	$2.8x10^{-6}$	$2.7x10^{-20}$	$2.7x10^{-20}$

<u>Supplementary Table 4:</u> *S. aureus* Viable Count Averages in single- and dual-species biofilm following antibiotic addition. Data from Figure 5. Biofilm was prepared as described in methods. Briefly, PBS washed ON cultures were normalised to 0.1 (commensals), =0.01 (S. aureus) and =0.001 (P. aeruginosa). After 24h, antibiotic (ciprofloxacin or tobramycin) was added at the desired concentrations and biofilms were further incubated 24h. After that time, viable count was performed. Excel statistics package was used to perform a t-Student comparing means among different conditions. A p-value ≤ 0.05 was considered statistically significant. **Ml**: *M. luteus*; **Pa**: *P. aeruginosa*; **Sa**: *S. aureus*; **Se:** *S. epidermidis*

**Species**	**Viable Count Mean (log_{10}CFUs/mL)**				***p*-values**		
**Ciprofloxacin Addition (µM)**	**0**	**16**	**64**	**256**	**16**	**64**	**256**
Sa	8.4±0.2	8.0±0.3	7.3±0.2	0.0	$1.3x10^{-6}$	$2.0x10^{-8}$	$9.5x10^{-64}$
Sa + Ml	6.7±1.5	6.7±0.5	6.4±0.4	5.8±0.5	0.053	0.075	$3.3x10^{-10}$
Sa + Pa	3.1±2.6	1.7±2.2	2.0±2.2	0.0	0.81	0.11	$1.81x10^{-4}$
Sa + Se	6.5±0.8	5.8±0.8	5.8±0.9	0.0	0.0079	0.0074	$5.0x10^{-28}$
**Tobramycin Addition (µM)**	**0**	**8**	**32**	**128**	**8**	**32**	**128**
Sa	8.4±0.2	7.1±0.5	6.1±1.8	3±3.1	$5.4x10^{-9}$	$7.4x10^{-5}$	$1.4x10^{-6}$
Sa + Ml	6.7±1.5	6.1±0.4	5.9±0.2	0.0	0.01	$3.5x10^{-4}$	$2.2x10^{-33}$
Sa + Pa	3.1±2.6	2.1±2.1	0.0	0.0	0.13	$3.3x10^{-10}$	$3.3x10^{-10}$
Sa + Se	6.5±0.8	5.2±0.3	0.0	0.0	$2.5x10^{-9}$	$5.0x10^{-28}$	$5.0x10^{-28}$

<u>Supplementary 5</u>: **Commensal Species Viable Count Averages in single- and dual-species biofilm following antibiotic addition.** Data from Figure 6. Biofilm was prepared as described in methods. Briefly, PBS washed ON cultures were normalised to 0.1 (commensals), =0.01 (S. aureus) and =0.001 (P. aeruginosa). After 24h, antibiotic (ciprofloxacin or tobramycin) was added at the desired concentrations and biofilms were further incubated 24h. After that time, viable count was performed. Excel statistics package XLSTAT was used to perform a t-Student comparing means among different conditions. A p-value ≤ 0.05 was considered statistically significant. **Ml:** *M. luteus*; **Pa**: *P. aeruginosa*; **Sa**: *S. aureus*; **Se**: *S. epidermidis*

**Species**	**Viable Count Mean (log_{10}CFUs/mL)**				***p*-values**		
**Ciprofloxacin Addition (µM)**	**0**	**16**	**64**	**256**	**16**	**64**	**256**
Se	8.6±0.3	7.9±0.2	7.6±0.1	0.0	$4.5x10^{-8}$	$1.3x10^{-22}$	$4.7x10^{-55}$
Se + Ml	8.1±0.2	6.9±0.6	6.3±0.2	0.0	$1.8x10^{-8}$	$2.1x10^{-28}$	$6.5x10^{-42}$
Se + Pa	1.3±2.0	0.0	0.0	0.0	0.0016	0.0016	0.0016
Se + Sa	8.0±0.3	7.5±0.1	6.8±0.3	1.8±2.6	$1.3x10^{-9}$	$2.1x10^{-16}$	$1.1x10^{-8}$
Ml	6.2±0.3	5.7±0.4	5.3±0.3	5.3±0.3	$5.0x10^{-5}$	$6.3x10^{-13}$	$1.3x10^{-14}$
Ml + Pa	5.8±0.8	5.5±0.04	5.2±0.3	4.5±0.2	0.052	$5.7x10^{-4}$	$2.2x10^{-11}$
Ml + Sa	0.0	0.0	0.0	0.0	N/A	N/A	N/A
Ml + Se	1.7±3.3	0.0	0.0	0.0	0.011	0.011	0.011
**Tobramycin Addition (µM)**	**0**	**8**	**32**	**128**	**8**	**32**	**128**
Se	8.6±0.3	7.4±0.2	6.8±0.1	5.8±0.3	$6.8x10^{-13}$	$3.0x10^{-24}$	$7.3x10^{-11}$
Se + Ml	8.1±0.2	5.9±0.3	6.0±0.2	3.9±0.1	$8.7x10^{-12}$	$2.0x10^{-18}$	$2.7x10^{-19}$
Se + Pa	1.3±2.1	0.0	0.0	0.0	0.0016	0.0016	0.0016
Se + Sa	8.0±0.3	6.7±0.2	6.6±0.3	6.7±0.1	$2.1x10^{-12}$	$4.7x10^{-8}$	$2.4x10^{-18}$
Ml	6.2±0.3	6.1±0.3	6.0±0.2	5.9±0.3	0.089	0.012	$1.0x10^{-4}$
Ml + Pa	5.8±0.8	7.3±0.2	7.3±0.1	7.1±0.1	$1.3x10^{-11}$	$2.5x10^{-12}$	$4.9x10^{-11}$
Ml + Sa	0.0	0.0	0.0	0.0	N/A	N/A	N/A
Ml + Se	1.7±3.3	0.0	0.0	0.0	0.011	0.011	0.011

<u>Supplementary Figure 1</u>: The dominance of *P. aeruginosa* visualised by cross-streak assay plate images. This is the 'raw data' which was ranked on a scale from dominant to suppressed to produce the data matrix displayed in Figure 3. To produce these plates: the primary species per plate was streaked four times horizontally (left to right), following by addition of each of the four species streaked vertically (top to bottom). A control, the species streaked across itself, is in the top left of each plate. Each plate represents a different primary species, (A) *M. luteus*, (B) *P. aeruginosa*, (C) *S. epidermidis* and (D) *S. aureus* These assays used the same 100:10:1 ratio of commensals: *S. aureus*: *P. aeruginosa* as the liquid growth experiments. The plates were incubated for 48-hours before photographing. Each plate was performed in triplicate, with a representative plate displayed.

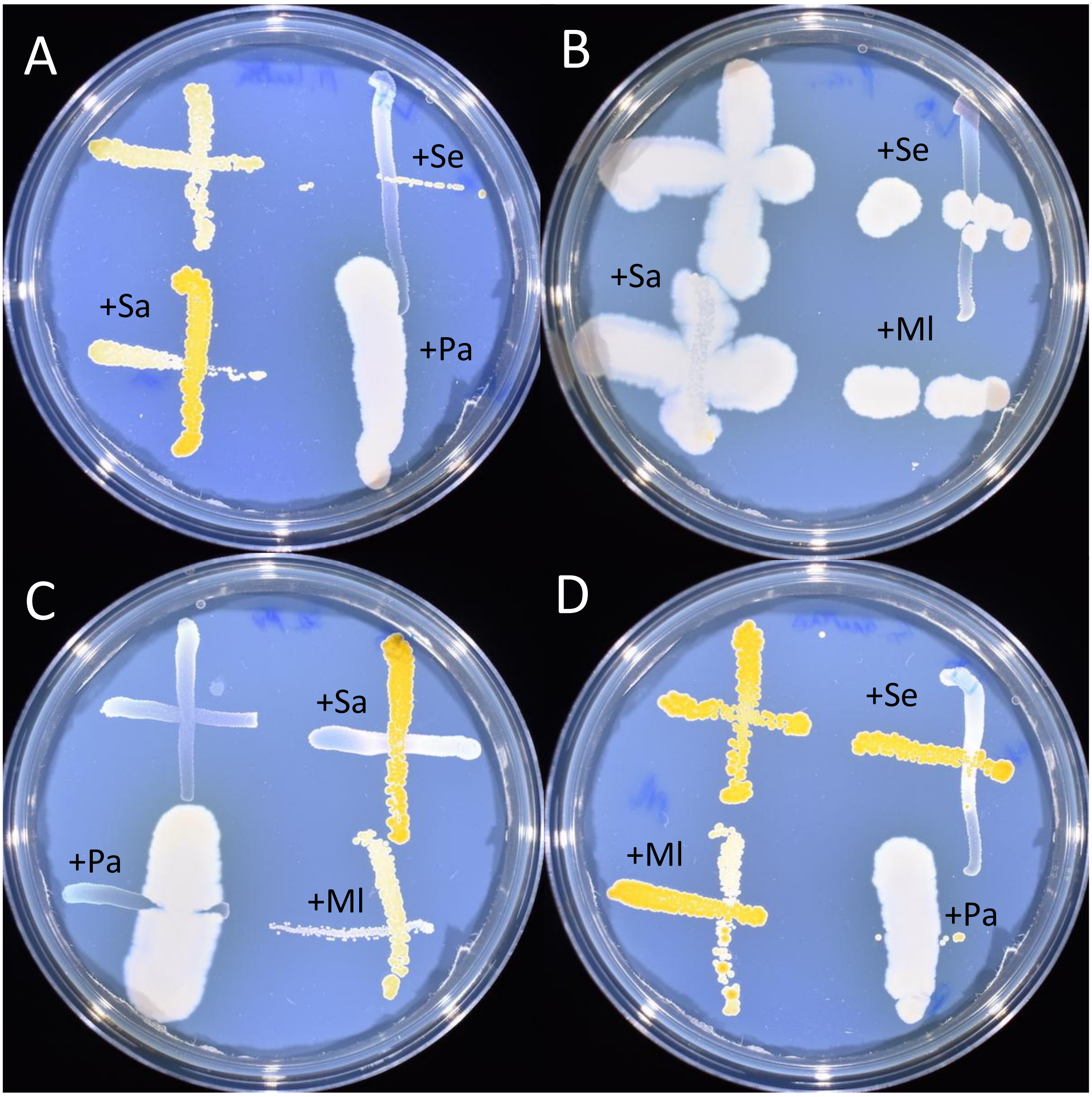

Supplementary Figure 2: Resistance to ciprofloxacin increased following biofilm formation. The graph indicates the MIC, MBC and MBEC of each species to ciprofloxacin, **Ml** = *M. luteus,* **Pa** = *P. aeruginosa*, **Sa** = *S. aureus* and **Se** = *S. epidermidis*. The MBC and MBEC values of ciprofloxacin against *M. luteus* could not be determined but were greater than 2,048 μM. Ciprofloxacin was bacteriostatic at low concentrations and killed each species at higher concentrations generating a gap between the MIC and the MBC. N=3

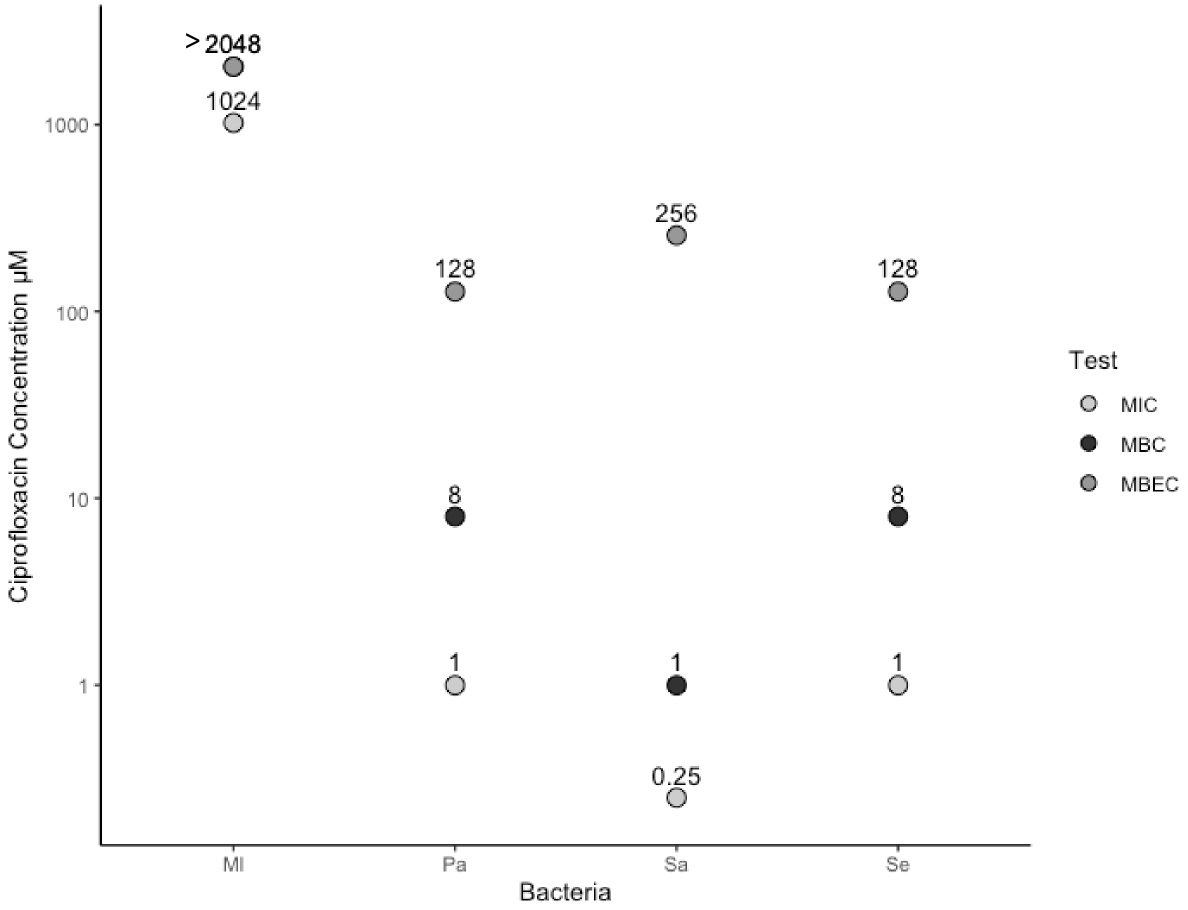

<u>Supplementary Figure 3</u>: Resistance to tobramycin increased following biofilm formation. Tobramycin appears to act bactericidally on all four species, as the MIC and MBC are similar or identical. The graph indicates the MIC, MBC and MBEC of each species to tobramycin**. Ml =** *M. luteus,* **Pa =** *P. aeruginosa*, **Sa =** *S. aureus* and **Se =** *S. epidermidis*. If the MBC value is not indicated, it is the same as the MIC. N=3

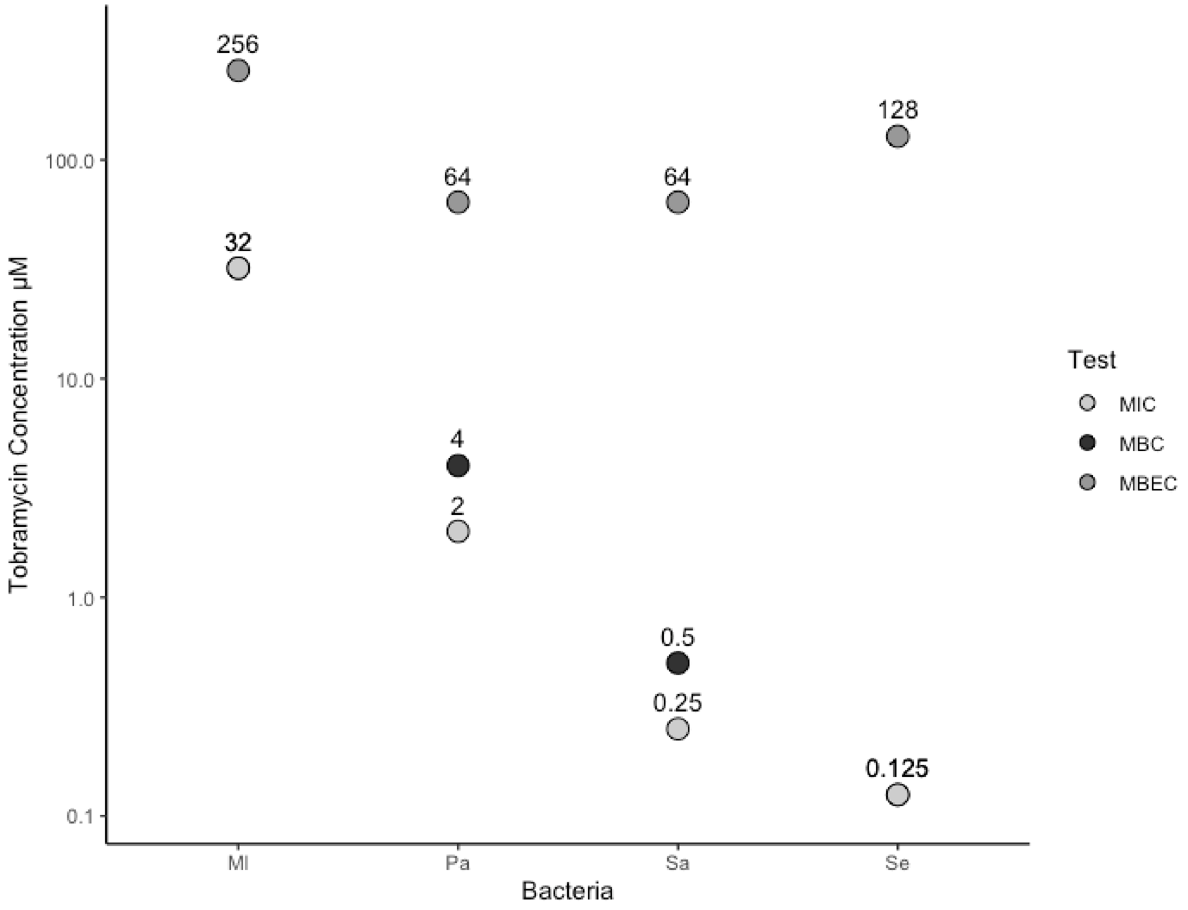

Supplement: Uncited Fig. S1. [file jmm-75-02126-s001.pdf]
